# Left ventricle reverse remodeling in chronic aortic regurgitation patients with dilated ventricle after aortic valve replacement

**DOI:** 10.1186/s13019-022-01754-5

**Published:** 2022-01-16

**Authors:** Ming-Kui Zhang, Li-Na Li, Hui Xue, Xiu-Jie Tang, He Sun, Qing-Yu Wu

**Affiliations:** Heart Center, First Hospital of Tsinghua University, No. 6 1st Street, Jiuxianqiao, Chaoyang District, Beijing, 100016 China

**Keywords:** Aortic valve regurgitation, Aortic valve replacement, Left ventricle remodeling, Follow up

## Abstract

**Background:**

Aortic valve replacement (AVR) for chronic aortic regurgitation (AR) with a severe dilated left ventricle and dysfunction leads to left ventricle remodeling. But there are rarely reports on the left ventricle reverse remodeling (LVRR) after AVR. This study aimed to investigate the LVRR and outcomes in chronic AR patients with severe dilated left ventricle and dysfunction after AVR.

**Methods:**

We retrospectively analyzed the clinical datum of chronic aortic regurgitation patients who underwent isolated AVR. The LVRR was defined as an increase in left ventricular ejection fraction (LVEF) at least 10 points or a follow-up LVEF ≥ 50%, and a decrease in the indexed left ventricular end-diastolic diameter of at least 10%, or an indexed left ventricular end-diastolic diameter ≤ 33 mm/m^2^. The changes in echocardiographic parameters after AVR, survival analysis, the predictors of major adverse cardiac events (MACE), the association between LVRR and MACE were analyzed.

**Results:**

Sixty-nine patients with severe dilated left ventricle and dysfunction underwent isolated AVR. LV remodeling in 54 patients and no LV remodeling in 15 patients at 6–12 months follow-up. The preoperative left ventricular dimensions and volumes were larger, and the EF was lower in the LV no remodeling group than those in the LV remodeling group (all *p* < 0.05). The adverse LVRR was the predictor for MACE at follow-up. The mean follow-up period was 47.29 months (range 6 to 173 months). The rate of freedom from MACE was 94.44% at 5 years and 92.59% at 10 years in the remodeling group, 60% at 5 years, and 46.67% at 10 years in the no remodeling group.

**Conclusions:**

The left ventricle remodeling after AVR was the important predictor for MACE. LV no remodeling may not be associated with benefits from AVR for chronic aortic regurgitation patients with severe dilated LV and dysfunction.

## Background

Aortic regurgitation (AR) involved both volume overload and pressure overload. The left ventricle (LV) adapts to the overloading by a series of compensatory changes, including LV dilatation and hypertrophy [1]. Therefore, patients with severe AR may tolerate the volume overload state for many years and remain asymptomatic even after the development of left ventricular dilatation and dysfunction [2]. Aortic valve replacement (AVR) is an effective treatment for patients with AR. In the current American Heart Association/American College of Cardiology guidelines and the European Society of Cardiology/European Society for Cardio-Thoracic Surgery guidelines, AVR is recommended for patients with severe chronic AR who have symptoms and/or LV dysfunction (ejection fraction, EF < 50%), LV end-diastolic diameter (LVEDD) > 65 or 70 mm, and/or LV end-systolic diameter (LVESD) > 50 mm [3, 4].

Several studies have investigated that AVR could correct the hemodynamic disturbance in AR patients with a significantly dilated LV and achieve postoperative LV reverse remodeling [5–7]. AVR is recommended for asymptomatic AR patients with LVEDD > 65 mm and normal LV systolic function [3, 4], but surgery sometimes results in persistent LV dilatation and systolic dysfunction, be associated with sudden death in follow-up periods [2]. The factors affecting LV reverse remodeling and outcome after AVR for severe LV dilatation and systolic dysfunction has not been researched in detail.

In the present study, we assessed the postoperative course after AVR for chronic AR patients with severe LV dilatation (LVEDD > 65 mm) and investigated the long-term prognostic impact of postoperative LV reverse remodeling.

## Methods

### Patients

The present retrospective study included 219 patients who underwent isolated aortic valve replacement for chronic AR from March 2004 to December 2018 at the heart center, First Hospital of Tsinghua University, China. Patients were excluded if they were undergoing previous aortic surgery, concomitant coronary artery bypass grafting, mitral valve repair or replacement, congenital aortic stenosis, acute infective endocarditis, aortic dissection. Perioperative death was defined as death within 30 days after AVR or in-hospital death. The left ventricular reverse remodeling (LVRR) was defined as an increase in LVEF at least 10 points or a follow-up LVEF ≥ 50%, and a decrease in the indexed left ventricular end-diastolic diameter of at least 10%, or an indexed left ventricular end-diastolic diameter ≤ 33 mm/m^2^, and LVRR was calculated based on baseline at 6 months to 1-year follow-up echocardiograms [8]. The severe LV dilatation was defined as left ventricle end-diastolic diameter (LVEDD) > 65 mm, preoperative left ventricle systolic dysfunction was defined as ejection fraction (EF) < 50% [3]. Based on the LVRR definition, these patients were divided into LV reverse remodeling group (remodeling group) and no reverse remodeling group (no remodeling group). The baseline clinical information and echocardiographic variables were retrieved from medical records. The study was approved by the ethics board of the First Hospital of Tsinghua University. Because clinical data was obtained in routine clinical practice, the informed consent from each patient was waived in this retrospective study.

### Surgical procedures

All patients have been treated with cardiotonic, diuretics, and other medications for heart failure before the operation. Hypothermic cardiopulmonary bypass and intermittent antegrade direct cold cardioplegia were routinely for the surgical procedure. There were 66 patients who underwent mechanical valve replacement, and 3 patients who underwent bioprosthetic valve replacement.

### Echocardiographic examination and parameters

Transthoracic echocardiography was performed before AVR, and at 6 months after AVR, using a commercially available ultrasound system (vivid7, E9) equipped with 3.5-MHz or M5S transducers. Two-dimensional and Doppler’s data were acquired at the parasternal, apical, subcostal, and supra-sternal views according to the standards of current recommendation. LV end-diastolic and LV end-systolic volumes (LVEDV and LVESV) were quantified in the apical two- and four-chamber views using Simpson’s biplane method, and LVEF was calculated according to the American Society of Echocardiography recommendations. Aortic valve function was evaluated using color, continuous- and pulsed-wave Doppler, AR was grade as 0 (absent), 1 (mild), 2 (mild-moderate), 3 (moderate-severe), or (severe) [9].

### Follow-Up

The major adverse cardiac events (MACE) included cardio-related death, hospitalization due to heart failure, and lethal arrhythmia. The follow-up data were obtained from our outpatients’ clinic records or by correspondence with referring physicians using telephone and subsequent hospitalization. The mean follow-up period was 47.29 months (range 6 to 173 months).

### Statistical analysis

Continuous variables were reported as mean ± standard deviation. The categorical variables were presented as numbers or percentages and compared using the Chi-square test or Fisher’s exact test. Continuous variables were compared using student’s t-test. The cumulative incidence of clinical outcomes after AVR was estimated using the Kaplan–Meier method. Multi-variable independent factors were performed by using stepwise regression. Statistical significance was defined as a 2-tailed *p* value < 0.05. All statistical analysis was performed with R version 3.5.3. (R Core Team (2019). R: A language and environment for statistical computing. R Foundation for Statistical Computing, Vienna, Austria. URL https://www.R-project.org/.)

## Results

Among these 219 patients, the study population consisted of 69 patients with preoperative severe LV dilatation or systolic dysfunction. Among these patients, the EF was less than 50% in 35 patients. The left ventricle end-diastolic diameter was between 65 to 70 mm in 34 patients, and it is between 70 to 80 mm in 26 patients, and more than 80 mm in 9 patients. The patients included 62 men (89.9%) and 7 women (10.1%) with a mean age of 47.06 ± 14.51 years (range 14 to 74 years). The mean body surface (BAS) was 1.87 ± 0.17 m^2^ (range 1.32 to 2.32 m^2^). Forty-one patients (59.4%) have class III or class IV heart failure, according to the New York Heart Association (NYHA) functional classification system.

Of these 69 patients who underwent isolated AVR, 54 patients with LV remodeling, and 15 patients with LV no remodeling comparing perioperative and early follow-up echocardiogram. The mean age at the AVR was 47.6 ± 15 years in the remodeling group, and 45.3 ± 12.7 years in the no remodeling group. The baseline characteristics and preoperative echocardiographic parameters of both groups were shown in Table 1. The time from diagnosis to surgery in the no remodeling group was longer than it in the remodeling group (*p* < 0.001). The echocardiographic parameters (LVEDD, LVESD, LVEDV, LVESV) were larger, and the EF was lower in the no remodeling group than those in the remodeling group (all *p* < 0.05).

**Table 1:** Baseline clinical and echocardiographic characteristics

Characteristics	Remodeling (n = 54)	No remodeling (n = 15)	*p* value
Male	48 (88.9%)	14 (93.3%)	1
Age at AVR	47.5 ± 15	45.3 ± 12.7	0.593
BSA (m^2^)	1.9 ± 0.2	1.9 ± 0.2	0.8
Hypertension	27 (50%)	5 (33.3%)	0.394
Diabetes mellitus	2 (3.7%)	1 (6.7%)	0.527
Coronary artery disease	7 (13%)	4 (26.7%)	0.237
Atrial fibrillation	4 (7.4%)	2 (13.3%)	0.604
Smoking	17 (31.5%)	8 (53.3%)	0.21
NYHA III/IV	29 (53.7%)	12 (80%)	0.124
Etiology of AR			0.057
Rheumatic	6 (11.1%)	3 (20%)	
Degenerative	18 (33.3%)	1 (6.7%)	
Congenital	17 (31.5%)	9 (60%)	
Other	13 (24.1%)	2 (13.3%)	
Time from diagnosis to surgery (years) surgery (months)	4.8 ± 6.2	15.4 ± 15.8	< 0.001
Echocardiographic parameters			
LVEDD (mm)	70.2 ± 4.6	80.6 ± 14.7	< 0.001
LVESD (mm)	51.7 ± 7.4	65.2 ± 18.4	< 0.001
LVEDV (ml)	230 ± 68.7	335.5 ± 149.2	< 0.001
LVESV (ml)	119.9 ± 51.3	203.1 ± 140.8	0.001
EF (%)	50.5 ± 10.6	37.7 ± 13.1	0.007
Procedure			1
Mechanical	51 (94.4%)	15 (100%)	
Bioprosthesis	3 (5.6%)		

*AVR* aortic valve replacement, *BSA* body surface area (m^2^), *NYHA* New York Heart Association, *AR* aortic regurgitation, *LVEDD* left ventricular end-diastolic diameter (mm), *LVESD* left ventricular end-systolic diameter (mm), *LVEDV* left ventricular end-diastolic volume (ml), *LVESV* left ventricular end-systolic volume (ml), *EF* ejection fraction (%)

**Fig. 1:**
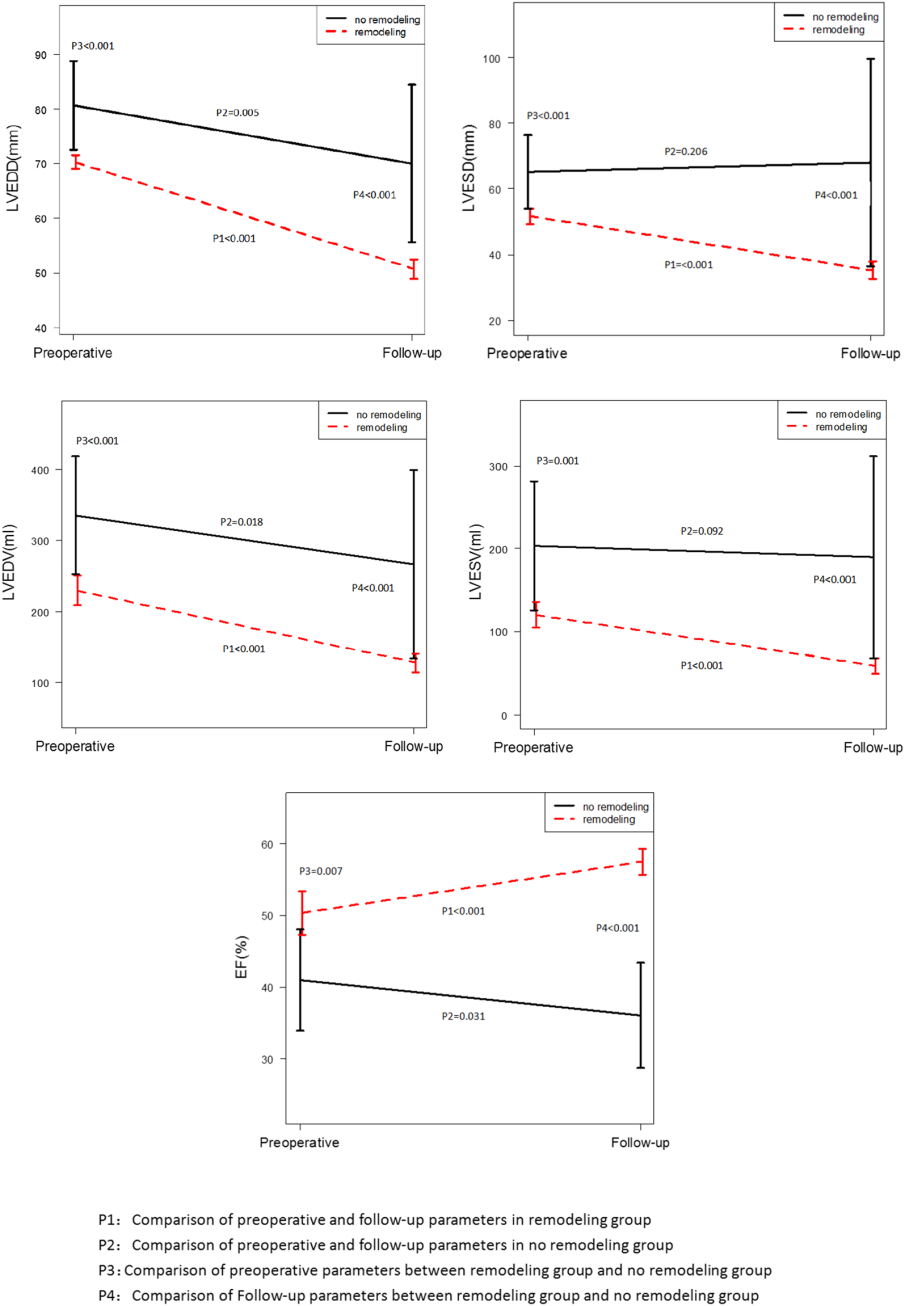
Comparing of the postoperative changes in echocardiographic parameters

Figure 1 shows the preoperative LV volumes, LV function, and follow-up results. In the remodeling group, the LVEDD, LVESD, LVEDV, and LVESV at follow-up were smaller than them at preoperative (all *p* < 0.01), and the LVEF increased during follow-up (all *p* < 0.01). But in the no remodeling group, the LVEDD and LVEDV at follow-up were reduced slightly compared with them at preoperative (all *p* < 0.05), but there were no significant changes in LVESD and LVESV during follow-up (*p* > 0.05). The multivariate stepwise regression analysis showed that no remodeling after AVR was a significant predictor for major adverse cardiac events (all *p* < 0.01) (Table 2).

**Table 2:** Predictors of major adverse cardiac events

Predictors	Crude OR (95% CI)	Adjusted OR (95% CI)	P (LR-test)
Time from diagnosis to surgery (cont. var.)	1.06 (1.1, 1.3)	1.05 (0.95, 1.18)	0.325
No remodeling versus remodeling	25.5 (5.38, 120.93)	58.68 (4.58, 751.62)	< 0.001
History of smoking (months)	1.06 (1.02, 1.11)	1.13 (1.02, 1.24)	0.004
Coronary artery disease: yes versus no	3.57 (0.85, 15.03)	1.42 (0.15, 13.4)	0.76

**Fig. 2:**
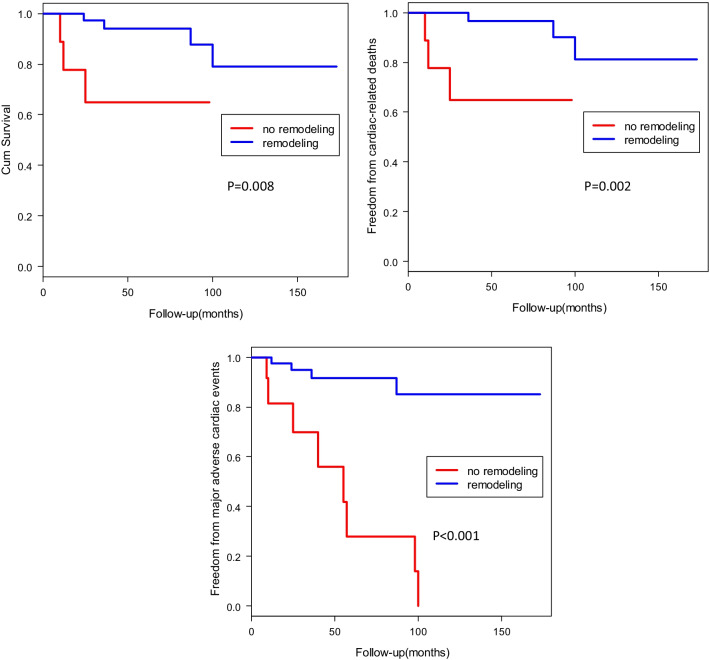
Kaplan-Meier curves for survival rate, the rate of freedom from the cardiac-related deaths, and MACE in the remodeling group and no remodeling group

The mean follow-up period was 47.29 months (range 6 to 173 months). During the follow-up period, there were 7 patients who died of all causes of death, the cause of death was non-cardiac-related in 1 patient, cardiac-related death in 6 patients. The cardiac-related events that occurred was 3 patients in the no remodeling group, there was 3 cardiac-related death cases in the remodeling group. The freedom rate from major adverse cardiac events (MACE), including cardio-related death, hospitalization due to heart failure, and lethal arrhythmia was 94.44% at 5 years and 92.59% at 10 years in the remodeling group, 60% at 5 years, and 46.67% at 10 years in the no remodeling group (Fig. 2). The survival rate from all-cause death in the remodeling group and no remodeling group were 96.30%, and 80%% at 5 years, 92.59%, and 80% at 10 years respectively (Fig. 2). There was a significant difference between the two groups (*p* = 0.008).

## Discussion

AVR is an effective treatment for patients with AR, which could achieve postoperative left ventricle remodeling. In present studies, the improvement of echocardiographic parameters after surgery has been shown in previous reports, but the left ventricle reverse remodeling after AVR for chronic aortic valve regurgitation patients with LV severe dilation and dysfunction was rarely reported [10–12]. Our findings demonstrated that LV reverse remodeling after AVR was observed in the majority of patients with ventricular dilation. AR associated with LV volumes overload and pressure overload, preoperative LVEF and left ventricle systolic dimension presented the pump performance, and left ventricle diastolic dimension presented the severity of volumes overload. Although AVR could correct the hemodynamic disturbance in AR patients, previous reports have suggested that the improvement of left ventricle function after aortic valve replacement early phase was due to the reduction of LV volume overloading [13, 14]. In this study, the mean LV diastolic dimension and LV ejection fraction were 70.2 mm and 50.5% in remodeling patients, but they were 80.6 mm and 37.7% for the no remodeling patients. We found that the postoperative LVEDD and LVEDV reduced significantly both in the remodeling group and no remodeling group, the characters were related to the correction of volumes overload after AVR. Unfortunately, the LVESD and LVESV in the no remodeling group had not been presented improvement after the surgery. The long-term pressure and volume overload might cause hemodynamic disturbance and pathological response in chronic aortic regurgitation could eventually result in myocardial fibrosis and irreversible left ventricle dysfunction [15]. Regeer and colleagues [5] reported that LV remodeling occurred both in patients with acute AR and chronic AR after AVR.

Guidelines for the management of chronic AR patients with normal left ventricle function were clear. The guidelines recommended that AVR was indicated for symptomatic patients with severe AR regardless of LV systolic function (Class of recommend 1), and for asymptomatic patients with severe AR and normal LV systolic function at rest (LVEF ≥ 50%) but with progressive severe LV dilatation (LV end-diastolic dimension > 65 mm) if the surgical risk is low (Class of recommend 2b) [3]. But the management of severe left ventricular dilation and dysfunction is challenging, conflicting results of long-term survival for these patients have been reported [13]. Some studies found that there was no significant difference in survival rate between cases with severe LV dysfunction and those with normal LV function after AVR [10, 16]. But a series of studies showed that 104 asymptomatic patients with normal LV systolic function followed for a mean of 8 years, and LV end-diastolic dimension of ≥ 70 mm was associated with a risk of death, symptoms, and/or LV dysfunction of 10% per year, and marked increases in end-diastolic dimension (≥ 80 mm) have been associated with sudden death [17]. We found that for the no remodeling patients with extremely enlarged ventricles, large volumes, and severe left ventricle dysfunction (the mean postoperative LVESD, LVESV, and EF were 61.7 mm, 179.2 ml, and 37.5% respectively), the LV systolic dimensions were not reduced after AVR. The LV remodeling after AVR is the predictor for long-term major adverse cardiac events. These changes might be presented that surgery could not influence the persistent LV dilation and dysfunction for LV no remodeling patients after AVR. Therefore, the clinicians should be aware that patients with these characters were high-risk and should not recover after AVR.

In this study, the results revealed that patients with LV remodeling after AVR had good clinical results than those patients with LV no remodeling. The results were thought to be due to myocardial changes in no remodeling patients at an irreversible stage. In the no remodeling group, more patients were rehospitalized due to cardiac deaths, lethal ventricular arrhythmia, or heart failure. Previous studies showed that cases with LVESD ≥ 55 mm and fractional shortening < 25% had a poor prognosis and more postoperative complication [18], extremely LV dilatation and markedly reduced EF means myocardial irreversible changes [19, 20].

## Limitations

There were several limitations to the study. First, this is a retrospective study, and not all of the patients could be followed up systematically, and resulting in a lack of complete datum in the long-term follow-up period after AVR. Second, as a single-institution study, the number of patients was relatively small, the small number of subjects might affect the statistical power of the variables.

## Conclusion

This study described that the left ventricle remodeling could occur in the majority of aortic regurgitation patients after AVR. However, the patients in the LV no remodeling group, with the characteristics of preoperative extremely left ventricle dilatation and markedly reduced EF, may not be associated with benefits from AVR due to postoperative lethal ventricular arrhythmia, heart failure, and poor long-term results.

## Data Availability

The data generated or analyzed during this study in this published article are available from the corresponding author on reasonable request.

## Abbreviations

AVR: Aortic valve replacement
AR: Aortic regurgitation
LVRR: Left ventricle reverse remodeling
EF: Ejection fraction
MACE: Major adverse cardiac events
LVEDD: Left ventricular end-diastolic diameter
LVESD: Left ventricular end-systolic diameter
LVEDV: Left ventricular end-diastolic volumes
LVESV: Left ventricular end-systolic volumes
